# Circadian timing of the dystrophin associated complex across the brain and body

**DOI:** 10.1186/s12987-026-00772-y

**Published:** 2026-02-12

**Authors:** Isaac Morse, Maiken Nedergaard, Lauren M. Hablitz

**Affiliations:** 1 Center for Translational Neuromedicine, University of Rochester Medical Center, Rochester, NY 14642 USA; 2 Center for Translational Neuromedicine, Faculty of Health and Medical Sciences, University of Copenhagen, Copenhagen, 2200 Denmark

## Abstract

The dystrophin associated complex (DAC) is an integral membrane scaffold that regulates cellular polarization and structural organization in both brain and peripheral tissues. In the brain, the DAC anchors the water channel aquaporin 4 (AQP4) to astrocytic vascular endfeet, thereby supporting glymphatic waste clearance during the inactive phase. Recent studies have raised the question of whether DAC gene expression is under circadian control. By mining four independent circadian transcriptome and translatome databases, covering multiple brain regions, peripheral tissues, ages, and species, we show that both *Aqp4* and the DAC components exhibit circadian rhythms in gene expression across most of the brain and body in mice, chickens and baboons. In addition, *Aqp1* shows circadian rhythmicity in peripheral tissues. The phase of these rhythms varies by tissue type yet collectively supports the hypothesis that clock-regulated DAC activity represents a conserved mechanism for coordinating ion, water, and structural homeostasis throughout the body.

**Supplementary Information:**

The online version contains supplementary material available at 10.1186/s12987-026-00772-y.

## Background

The glymphatic system is a brain-wide perivascular fluid pathway that enables cerebrospinal fluid (CSF) influx into the brain, facilitating interstitial fluid exchange to remove metabolic waste, including lactate and peptides such as amyloid beta [1–5]. Glymphatic activity is controlled by circadian timing, with greater CSF influx and solute clearance during the inactive phase, and enhanced drainage to the cervical lymph nodes during the active phase [6]. Understanding how circadian mechanisms regulate glymphatic function is critical, as glymphatic dysfunction has been implicated in aging and a wide range of neurological disorders [4, 7–11], many of which are also associated with circadian dysruption [12–15]. 

Circadian rhythms of glymphatic function and lymphatic drainage depend on the water channel aquaporin 4 (AQP4), which is enriched at the vascular endfeet of astrocytes [6]. This localization is maintained by the dystrophin-associated complex (DAC), a large scaffolding assembly that anchors AQP4 in astrocytes and regulates protein distribution throughout the central nervous system, muscle, and peripheral tissues [16, 17]. Previous studies support daily regulation of the DAC and AQP4, showing diurnal variation in mRNA expression of both AQP4 and DAC components [6]. Loss of BMAL1, a core clock transcription factor, disrupts AQP4 expression [18] reinforcing the hypothesis that these genes are clock-controlled. However, the extent of this regulation remains debated, with one recent publication reporting no day/night differences in DAC and AQP4 in restricted regions of cortex and hippocampus [19]. There are multiple potential reasons for the discrepancies between these studies, including limited time-series sampling and known brain-region specific differences in amplitudes of circadian gene expression [20–22]. Thus, it is necessary to further investigate whether the DAC is under circadian control.

Understanding how circadian timing influences the DAC has broad implications not only for glymphatic function but also for fluid homeostasis and tissue architecture across the body. To directly address whether mRNA for the DAC and AQP4 exhibits circadian regulation, we analyzed four large databases of circadian gene expression in the brain and peripheral tissues [23–26]. Our analysis revealed significant rhythmicity of DAC components in most tissue under constant dark conditions, supporting the conclusion that both DAC and AQP4 are under circadian control.

## Methods

### Data collection

Data on circadian rhythmicity, acrophase (peak transcription time of significant rhythms) and differential rhythms in tissues was collected from 3 published transcriptomes databases (“DB”, http://circadb.hogeneschlab.org/mouse [23]; “Age”, https://circaage.shinyapps.io/circaage/ [24]; “KB”, https://cdsic.njau.edu.cn/CircaKB/#/Home [25]) for a total of 12 time course data sets of C57BL/6 mice in constant darkness. 9 of the 12 data sets were analyzed using JTK_CYCLE analyses for determining rhythmicity and acrophase. 3 datasets from the CircaAge database utilized cosinor analysis to determine rhythmicity and acrophase, and a likelihood-based test with a *p* value < 0.01 for significance. For all datasets that did not have Rao’s spacing or Rayleigh tests, data was included only if *p* < 0.05.

An additional 3 studies from CircaKB were used to investigate if the rhythmicity of DAC members and their putative binding partners is conserved across species (chicken and baboon). All studies used wild type animals of the given species on a 12:12 light: dark cycle but were subject to the same criteria above. Tissues sampled from the brain include the retina and choroid for Chicken and Baboon, as well as the brainstem, cerebellum, prefrontal cortex and SCN in only the baboon. Body tissues sampled from the baboon include the heart, lungs, multiple skeletal muscles and the medulla and cortex of the kidneys.

Data from publicly available translatome time courses [26] were analyzed for time-of-day differences in ribosome associated mRNA. This data was taken from 2 wild-type or, for astrocyte specific RNA, Aldh1l1:EGFP/RPL10A mice every 2 h for a total of 24 h under constant darkness.

### Exclusion criteria

Any study that performed significant surgery before tissue collection, or that separated timepoints by greater than 5 h, were excluded from this study. For the comparison of chicken and olive baboon rhythms, *Mlc1* was only reported in the retina of olive baboons [27] where it was arrhythmic. All other genes were expressed in both species, in at least two different tissues, thus *Mlc1* data was not included in the figure.

### Animal information

From the CircaDB database [23], mouse data was pulled from four separate studies. Two used male C57BL/6 mice approximately 6–8 weeks old [28, 29]. One study, which provided data for muscle, did not specify sex of the mice but used C57BL/6 mice approximately 12–14 weeks old [30]. The final study did not specify age, sex, or genetic background of the mice used in the study [31]. For the Circa KB database [25], there were four mouse studies. One used male and female C57BL/6J mice 6–7 weeks old [32]. Another used 7–10 weeks old male C57BL/6J mice [33]. The final two datasets were from C57BL/6 mice that did not have age or sex specified (Gene Expression Omnibus numbers: GSE70384 and GSE70391). Data from the translatome database was generated from 6 to 6.5 week old male and female mice in equal numbers [26]. For data from the CircaAge database, male C57B6/J-NIA mice at 6mo and 18mo were treated as two “adult” time courses, “old” mice were 27 mo [24]. The male and female chickens (*Gallus* gallus) used were Cornell-K closed flock subjects that were 2 weeks old [34]. All baboons (*Papio* *anubis*) were male and between 6 and 8 years old [27]. 

### Calculation of percent rhythmicity

Those rhythms that were statistically significant using JTK_cycle (from CircaDB or CircaKB), or cosinor analysis (CircaAge) were then divided by the total number of datasets available. For example, there were four datasets reporting information for AQP4 in the cerebellum. Of those four, all had a statistically significant JTK_cycle value in the CircaDB database. Thus, rhythmicity is 100%. A low percentage rhythmicity does not mean the gene is arrhythmic. JTK_cycle is somewhat unreliable for detecting non-parametric rhythms in time courses with low animal number, and cosinor analyses are frequently considered overly-conservative in the field because of the dependency on a sine wave function [35, 36], so any time course that has a significant statistical value is meaningful. Please see Fig. S1 for the total number of time courses taken for each tissue.

### Data visualization

Acrophases were collected by tissue, then gene, and graphed using R Studio (Rayleigh/Circular plots) or GraphPad Prism9.

### Rao’s spacing and Rayleigh tests

For the Rao’s spacing and Rayleigh tests in Fig. 2d and e, any *p* value < 0.1 were included to ensure appropriate sample sizes to run these tests. Rao’s spacing test and Rayleigh tests were run to determine the uniformity of peak time distributions using the R Studio package “circular”. Additionally, to get an estimate of the numerical *p* value from the Rao’s spacing test, a 10,000 sample Monte Carlo simulation was run using the same data in R Studio [37].

## Results

### The DAC and *Aqp4* exhibit circadian gene expression in most tissue

**Fig. 1:**
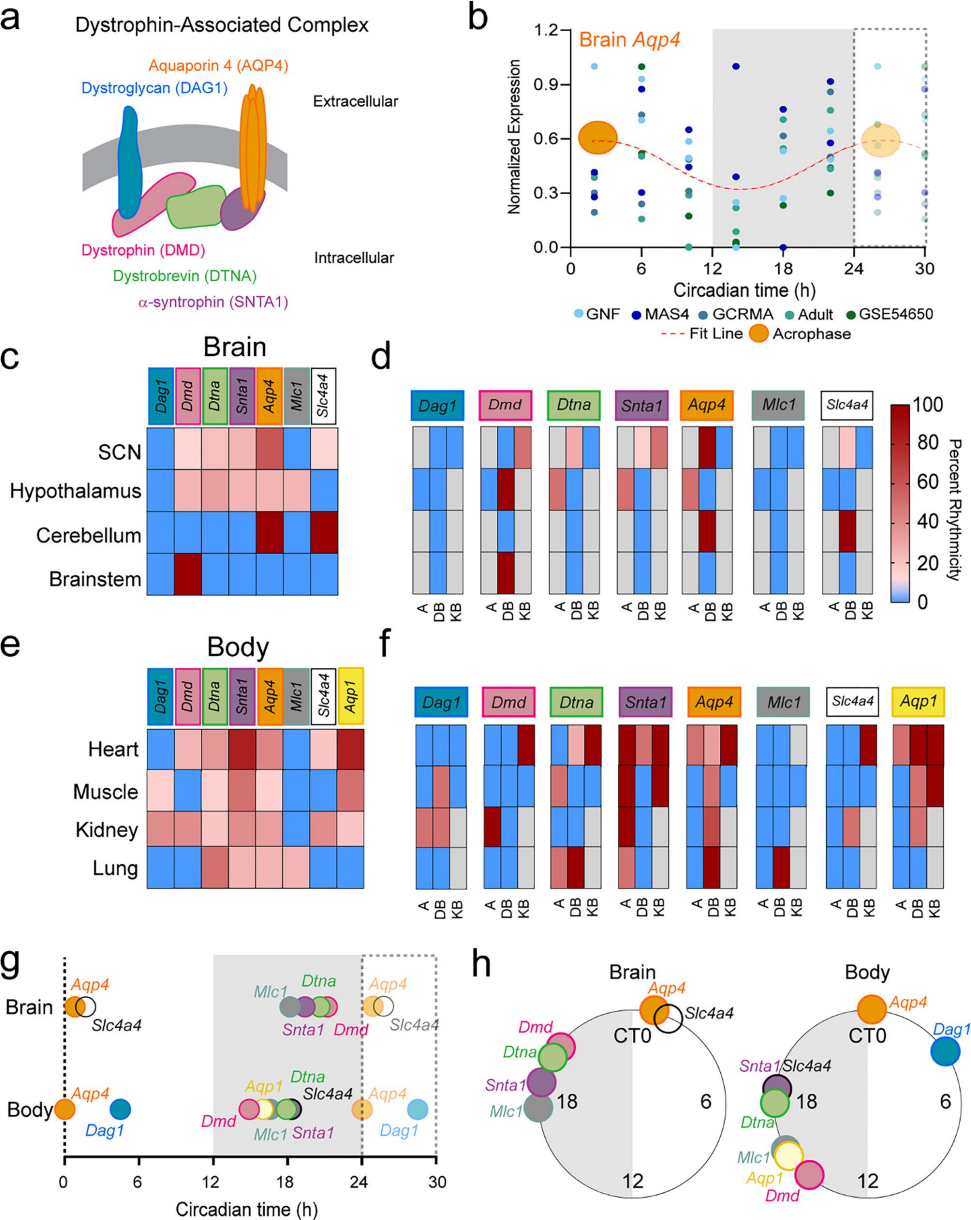
Gene expression of the DAC exhibits circadian timing across the brain and body. (**a**) A schematic of the dystrophin associated complex. (**b**) Representative scatter plot of RNA-seq data of gene expression for *Aqp4* across brain regions. Individual dots are colored for the dataset represented, with each dot a single mouse. Circadian time indicated in hours, where Circadian time 12 is activity onset in mice. A best fit sine wave is shown in red as an example of how to determine if the data is rhythmic. Note: a best fit line was shown for this cumulative plot of multiple datasets for ease of viewing a rhythm, but this is not a test of rhythmicity. All data was analyzed using circular statistics like JTK_cycle or cosinor analysis as specified in the methods. Average acrophase of *Aqp4* expression across all brain datasets shown in orange. The lighter colored region from 24–30 hours is a replot of earlier data to help visualize rhythmicity. (**c**) Percent rhythmicity of different genes across databases in specific brain regions. (**d**) (left) The data from (**c**) broken down by database. A, CircaAge; DB, CircaDB; KB, CircaKB. Gray boxes indicate no results from the database. (right) Percent rhythmicity scale bar. (**e**) Same as (**c**), but in different tissues throughout the body. (**f**) Same as (**d**) but for body tissues. (**g**) Average acrophase of each gene across brain and body regions across the circadian day. The lighter colored region from 24–30 hours are repeats of earlier data to better portray the cyclic nature of transcription. To visualize the data for circular analysis of clustering of acrophases, data from g is plotted in (**h**), a Rayleigh plot of the average acrophase of each gene across brain and body regions. Gray regions depict the dark, active phase in mice. Individual genes represented by colored dots around the 24h plot

First, we confirmed rhythmicity of the circadian molecular clock components *Arntl* (*Bmal1*), *Per1* and *Per2* across tissues as a positive control, and a lack of rhythmicity in *Arnt*, a homologue of *Arntl*, as a negative control as previously reported (Fig. S1) [38]. Next, we investigated whether there were rhythms in the transcription of the DAC (*Dag1*, *Dmd*, *Dtna*, *Snta1*) and putative binding partners (*Aqp4*, *Mlc1*, *Slc4a4*) in the brain and body (Fig. 1). In the brain, datasets included the suprachiasmatic nucleus (SCN, a small hypothalamic nucleus considered the clock center of the brain [39]), the hypothalamus, the cerebellum, and the brainstem. Direct analysis of rhythmic DAC components in whole brain, cortex, and hippocampus were not available in these datasets, limiting direct comparisons with previous results [6, 19]. Overall, more genes were rhythmic in the SCN and hypothalamus compared to brainstem and cerebellum (Fig. 1c, d), reinforcing the hypothesis that brain regions of hypothalamus exhibit higher amplitude, more synchronized rhythms compared to cerebellum or brainstem where the amplitudes may be damped or impacted by arousal state [22, 40]. *Dmd* and *Aqp4* exhibited significant circadian rhythmicity across three out of four brain areas (*Dmd*: SCN, hypothalamus, and brainstem; *Aqp4*: SCN, hypothalamus, and cerebellum). *Slc4a4* was rhythmic in the SCN and cerebellum, while *Dtna* and *Snta1* were both rhythmic in SCN and hypothalamus. Out of all the DAC, *Dag1* was the only completely arhythmic gene measured across selected brain tissues. We conclude that both the DAC and putative binding partners including *Aqp4* and *Scl4a4* exhibit significant circadian rhythmicity in gene expression that is brain-region specific.

The DAC is not only in the brain but acts as a scaffold complex throughout the body, playing a crucial role in tissue polarization in heart, kidney, lung, muscle, and more [41]. In light of this, we expanded our study of DAC rhythmicity to peripheral tissues (Fig. 1e, f). In contrast to the brain, *Dtna*, *Snta1*, and *Aqp4* were all highly rhythmic across body tissues (heart, kidney, lung, muscle). *Aqp1* was rhythmic in all tissue except lung. *Dag1*, *Dmd*, and *Slc4a4* were rhythmic in two out of four tissues, though the tissue types were variable. Finally, *Mlc1* was only rhythmic in the lung. In the kidney, every gene investigated exhibited circadian rhythmicity. Heart also exhibited significant rhythmicity except for *Dag1* and *Mlc1.* Lung was the most variable tissue, with 4 out of 8 genes demonstrating circadian rhythmicity. Based on this analysis, *Aqp4* and the DAC expressed circadian rhythmicity not only in the brain but throughout the body in a tissue-dependent manner.

### Rhythms in *Aqp4* gene expression peak after coordinated DAC gene expression

**Fig. 2:**
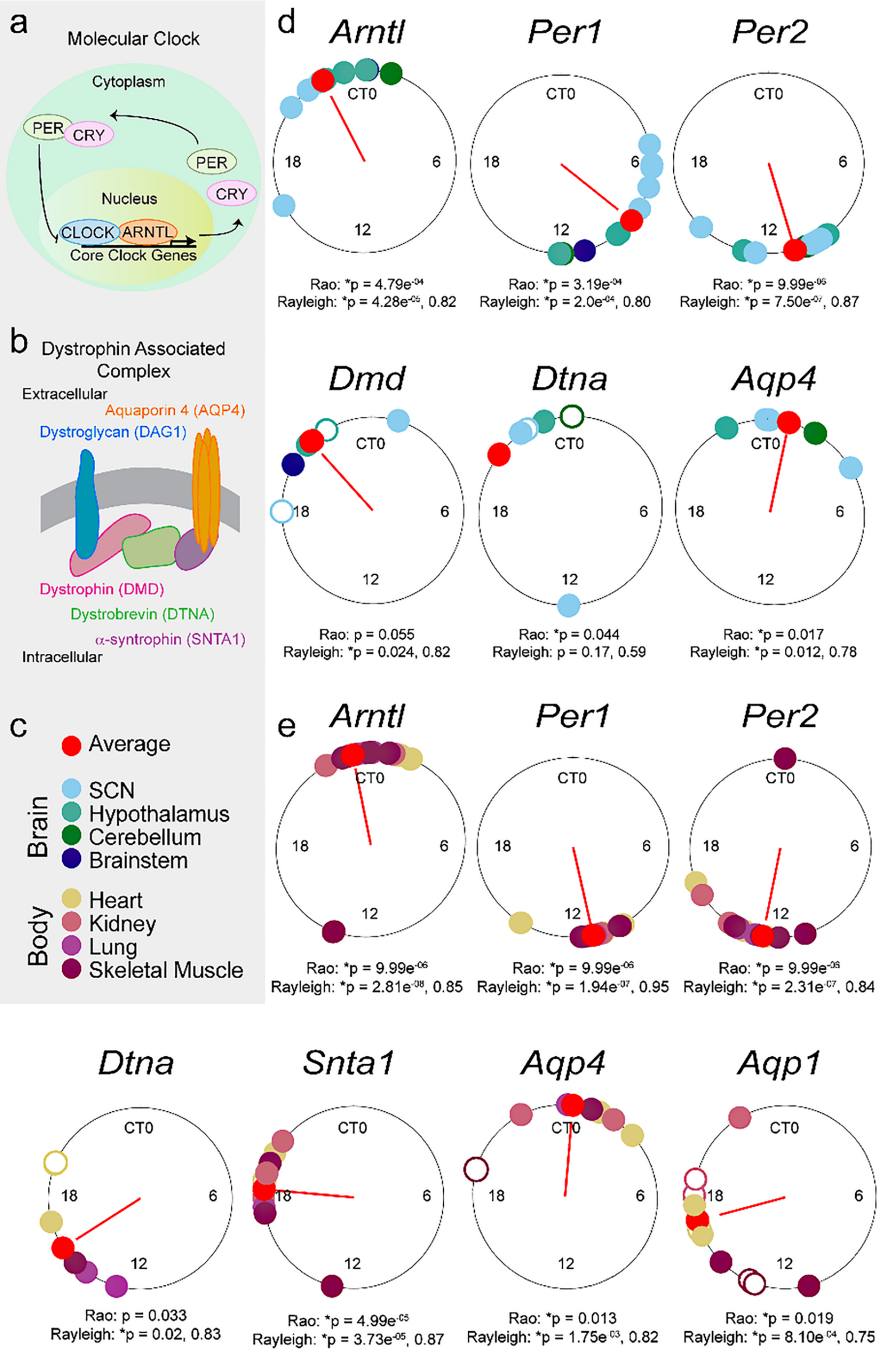
Gene expression of the DAC precedes putative binder expression across the day. Gray box indicates reference information for this figure. (**a**) A schematic of the circadian molecular clock. (**b**) A schematic of the Dystrophin-Associated Complex. (**c**) Color key for brain and body regions represented on the Rayleigh plots. Note, if there was more than one significant time course for each tissue there are more than one similar colored dots on the Rayleigh plot. (**d**) Rayleigh plots of gene/tissue acrophases. Empty circles represent the acrophase of trends (*p* < 0.1). Red lines are vector plots of Rayleigh test value for average timing of acrophase (red circle). P values and test statistics are reported for Rao and Rayleigh tests under each plot. (**e**) Same as (**d**) but from tissues in the body

Circadian rhythms in DAC gene expression should synchronize to ensure timed tissue polarization. To test this, peak time of gene expression, also known as acrophase, was plotted for all rhythms both as an average across brain and body tissues (Fig. 1g, h) and in Rayleigh plots organized by tissue type (Fig. 2). Consistent with previous literature [23–25], *Arntl* peaked at Circadian Time (CT, CT 12 is activity onset) CT 22, while *Per1* peaks at CT 8.6 in the brain. There is some variability, most likely due to the wide range of brain areas sampled (brainstem, cerebellum, hypothalamus, and SCN).

Glymphatic function peaks at CT 6 [6]. If AQP4 supports this rhythm, gene expression rhythms should peak in the early inactive phase. Indeed, *Aqp4* peaks at CT 0.85, about 50 min into the inactive phase, consistent with increased glymphatic function (Fig. 1g, h). *Slc4a4* also peaks around this time at CT 1.7. Interestingly, DAC expression largely precedes this uptick in *Aqp4* expression (*Dmd*, CT 21.2; *Dtna*, 20.6; *Snta1*, CT 19.4), supporting the hypothesis that coordinated DAC/AQP4 expression supports circadian rhythms in brain fluid homeostasis. In contrast, *Mlc1* peaks immediately prior to the DAC (CT 18.2), indicating this gene may not play a role in glymphatic fluid movement.

Similar to brain tissue, body *Aqp4* expression peaks at the beginning of the inactive phase (CT 0.21). Yet, the DAC no longer directly precedes *Aqp4* expression (*Dmd*, CT 14.9; *Dtna*, CT 17.9; *Snta1*, CT 18.3; *Dag1*, CT 4.5). Instead, *Slc4a4*,* Mlc1*, and *Aqp1* become more in phase with the DAC (*Slc4a4*, CT 18.3; *Mlc1*, CT 16.6; *Aqp1* CT 16), indicating that the DAC may have a temporally conserved role in the driving water and ion homeostasis in the brain and body using different putative binding partners.

To better understand the timing of gene expression between brain regions and across tissues, we performed Rao and Rayleigh tests on the acrophases of gene expression rhythms across brain or body tissue for each gene. The Rao test asks if there is non-normal clustering of datapoints, where the Rayleigh test asks if there is significant clustering at a specific time [37, 42]. As expected, core clock genes exhibited significant Rao and Rayleigh tests in both the brain and body (Fig. 2d, e), with windows of gene expression up to 10 h apart. This 10-hour window reflects the time it takes the SCN to synchronize molecular clock rhythms in the brain and body, driving variability in expression of clock-controlled genes across cell and tissue types [43–45]. Because some of the sample sizes were low there is a limit to the power of these analyses. Despite this, in the brain, *Dmd* and *Dtna* showed trends of clustering, peaking 2–6 h before CT 0. *Aqp4* had significant Rao and Rayleigh tests, with a peak in the early inactive phase after *Dmd* and *Dtna*. The body had a greater number of genes with significant Rao and Rayleigh tests, with *Dtna*,* Snta1*,* Aqp4*,* and Aqp1* all having significant clustering (Fig. 2).

### The translatome exhibits time-locked DAC expression

**Fig. 3:**
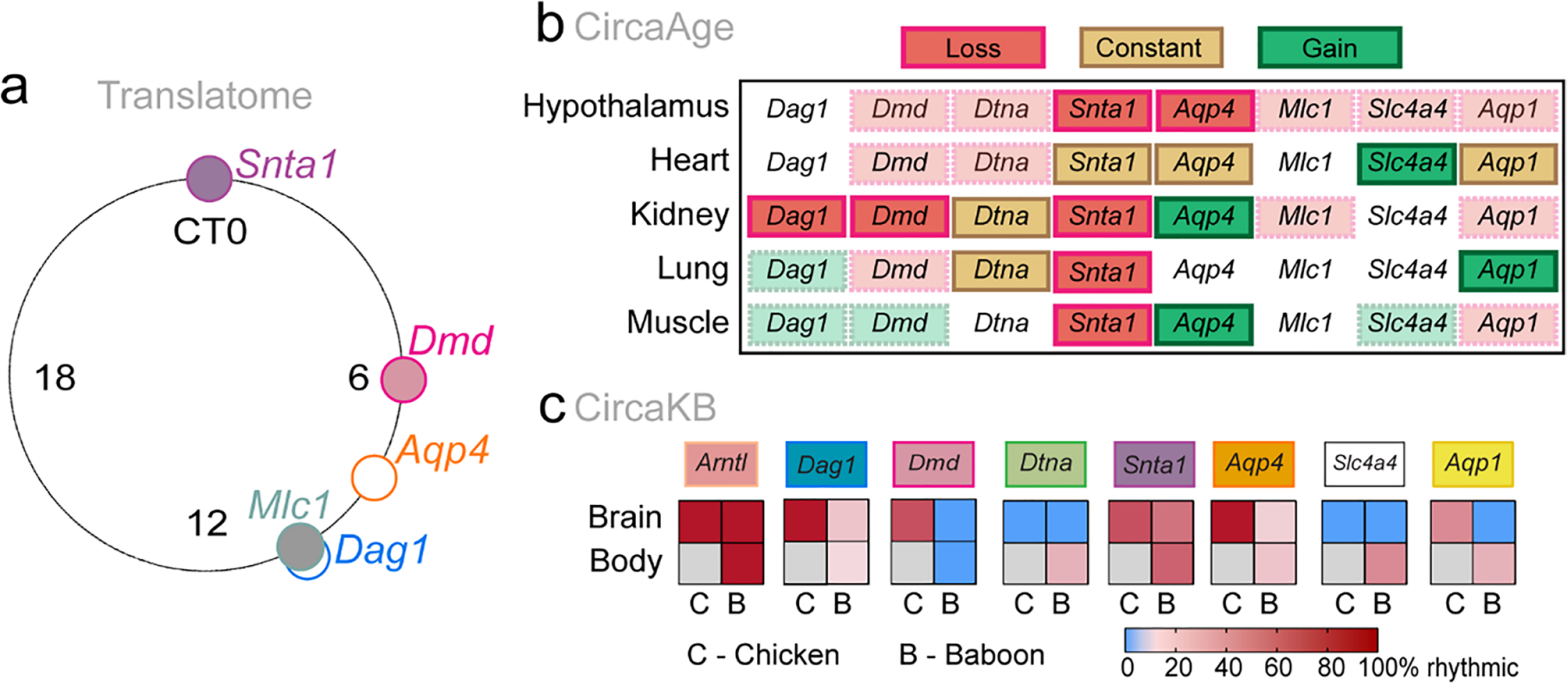
DAC expression is regulated by circadian timing through translation, across age, and across species. (**a**) A Rayleigh plot of the translatome data depicting gene translation acrophases within the brain. Empty circles represent the acrophase from studies that reported trends (*p* < 0.1). (**b**) Data from CircaAge. A table depicting genes which either lost (red) or gained (green) rhythmicity as mice age. Genes that were consistently rhythmic are in gold. Opaque colors indicate genes which had trends in either youth or age but not the other. Loss, constant, or gain was determined by significant *p* values from a cosinor analysis between young/middle aged mice compared to old mice. (**c**) Data from CircaKB. A heatmap of gene rhythmicity for alternative model animal species (Chicken, *Gallus gallus* and Baboon, *Papio anubis)* across the brain and body. Color bar of percent rhythmicity in the lower right

Rhythms in ribosome-associated mRNA [26] were investigated for core clock genes, the DAC, and putative binders to test whether the rhythms found in overall gene expression continued to mRNA being translated (Fig. 3a). Core clock gene rhythmicity in the brain has already been published for this dataset [26]. Significant rhythms *Snta1*, *Dmd* and *Mlc1* were found, with trends in *Aqp4* (*p* = 0.058) and *Dag1* (*p* = 0.060). Interestingly, the acrophases of these rhythms followed what was found in the transcriptome datasets, with *Snta1* and *Dmd* preceding *Aqp4* and *Mlc1*, suggesting that both gene transcription and translation of the DAC are under clock control.

### Circadian rhythmicity changes with aging

We next examined how the rhythmicity of the DAC, *Aqp4* and *Aqp1* changed from young/middle aged to old mice within the hypothalamus, heart, kidney, lung and muscle (Fig. 3b) using the CircaAge database [24]. Specifically, we looked for which genes exhibited significant cosinor *p* values at younger ages compared to the old cohort. Overall, *Snta1* was rhythmic in all young tissue, and lost rhythmicity with age in 4/5 tissues, indicating this gene may play a role in age related changes to water and ion homeostasis. Three genes consistently showed loss of rhythmicity across tissues with aging (*Dag1*,* Dmd*,* Snta1*) while gains of rhythmicity were seen in *Aqp4*,* Slc4a4*,* and Aqp1* in a tissue specific manner, perhaps evidence of molecular clock programming changing with age [46]. The heart was most protected against the effects of aging with *Snta1*, *Aqp4*, and *Aqp1* exhibiting circadian rhythms at both ages. The brain lost rhythmicity in 2/8 DAC and associated genes, with a trend in a further 5/8, coinciding with the observation that glymphatic function decreases with age [1].

### Daily rhythms in DAC expression are observed in multiple species

In mice, DAC expression is regulated in a circadian manner across the brain and body, changing with age. We hypothesize that these daily rhythms persist across species because of the evolutionarily conserved nature of circadian gene regulation [47, 48], and the fundamental need for fluid homeostasis in biological tissues. Indeed, in chicken brain tissue, 5/7 genes show rhythmicity (*Dag1*, *Dmd*, *Snta1*, *Aqp4*, and *Aqp1*) and in the brain tissue of the olive baboon, *Papio anubis*,* Dag1*, *Snta1*, and *Aqp4* all show daily rhythms (Fig. 3c). Rhythms in the DAC are more apparent in the peripheral tissue from the olive baboon, with rhythms in 6/7 genes investigated (*Dag1*, *Dtna*, *Snta1*, *Aqp4*, *Slc4a4*, and *Aqp1*). These data demonstrate there are daily rhythms to DAC expression and *Aqp4* that may support changes to fluid homeostasis in the brain and body across the day in multiple species.

## Conclusions

Using large, pre-existing circadian datasets, we demonstrate that the DAC and *Aqp4* exhibit significant circadian rhythms in gene expression across most brain and body tissues. In the brain, the DAC components peak earlier than *Aqp4*, which is most highly expressed during the inactive phase, supporting the hypothesis that the DAC promotes AQP4 polarization and, by extension, glymphatic flow. Rao and Rayleigh tests of acrophase distributions revealed temporal expression patterns that varied by gene and tissue type, consistent with the multi-oscillator architecture of circadian biology. Importantly, rhythmicity was not limited to *Aqp4* but also included *Mlc1*,* Slc4a4*, and *Aqp1*, established DAC binding partners and water channels, suggesting a conserved circadian mechanism for DAC-mediated regulation of water and ion homeostasis.

Conflicting results from prior studies likely reflect methodological limitations. One study reported day/night differences in *Aqp4*, *Dag1*, and *Dtna* using total brain homogenates [6], while another observed inverted day/night differences in micro-dissected parietal cortex and hippocampus for *Dag1*, *Slc4a4*, and *Snta1*, and no rhythmicity in *Aqp4* [19]. Both studies relied on only two timepoints (midday and midnight), precluding valid assessment of circadian dynamics. By contrast our analysis of four large-scale circadian transcriptional databases, representing over 15 independent course datasets, shows that DAC and *Aqp4* expression consistently peak in the late active to early inactive phase with variation in rhythmicity and timing across brain regions, providing a unifying explanation for discrepancies in earlier reports. This rhythm in gene expression is also consistent with functional output of the glymphatic system, namely increased CSF influx and solute clearance from the brain observed in the inactive phase of the mouse [6]. 

In mammals, circadian rhythms are synchronized by the suprachiasmatic nucleus of the hypothalamus, which coordinates time cues to the brain and body through neuronal connectivity and hormonal signaling [39]. This organization produces phase lags between peripheral oscillators, reflected in this study by the spread in acrophases for molecular clock markers *Arntl* and *Per*. Based on this information, it is unsurprising, anticipated even, that DAC and *Aqp4* rhythms are not perfectly aligned across brain regions, an observation with implications for glymphatic function. In humans, neuronal activity rhythms peak across cortical regions over a four-hour window [49], and synchronized neuronal activity has been proposed as a key driver of glymphatic flow [50, 51]. Together, these findings suggest that circadian variation in both neural activity and DAC expression may guide fluid transport along specific anatomical pathways to promote efficient solute clearance.

Circadian rhythmicity of the DAC was also observed in the heart, kidneys, lungs, and skeletal muscle. In the heart, aquaporins are important for edema regulation following ischemia–reperfusion injury [52], through their role in contractility and electrical conduction remains poorly understood [53]. In the kidney, circadian control of glomerular filtration and tubular secretion/reabsorption involves AQP4 [54, 55], which is also essential for collecting duct function [56]. In the lungs, deletion of AQP1 severely impairs fluid transport and reduces interstitial flow [57]. Interestingly, there is SCN-driven regulation of hydration state and kidney function driven primarily by vasopressin signaling [58]. It is tempting to speculate that the SCN-vasopressin axis may be regulating circadian timing of DAC and aquaporin gene expression. Collectively, these findings indicate that circadian rhythmicity in DAC, aquaporins, and solute carriers represents a conserved mechanism for regulating fluid dynamics across both brain and body.

Future work may explain how, exactly, daily rhythms in the DAC may support fluid homeostasis. For example, how does temporal niche impact fluid homeostasis? Though SCN rhythmicity and systemic melatonin is consistent across temporal niches, behavior and metabolism can niche-switch [59–61]. Furthermore, in the brain, fluid dynamics are driven by perivascular flow, with AQP4 localized to the vascular endfeet of astrocytes supporting this movement [3, 62, 63]. How does this work in peripheral tissue without perivascular localized glymphatic flow? And finally, despite these newly described endogenous, circadian rhythms in mRNA levels the DAC across the brain and body, how this translates to protein levels and localization remain underexplored. Previous work has demonstrated that in whole brain homogenate total levels of AQP4 do not exhibit day/night differences, though splice variants may vary [6]. This lack of day/night difference was confirmed in parietal cortex and hippocampus [19]. Day/night differences in localization of AQP4 is variable across brain regions [6, 19]. There is a wide body of literature that suggests both gene expression and posttranslational modifications are under clock-control [64, 65], and may explain some discrepancies in the published literature of AQP4 and the DAC. While circadian timing mechanisms are conserved across tissue type, levels of biological organization, and species – the exact mechanisms may change depending on biological need [47, 48]. How these concepts impact the glymphatic system, or fluid and ion homeostasis across biological tissues at large, remain to be explored.

## Supplementary Information

Below is the link to the electronic supplementary material.

Supplementary Material 1

## Data Availability

All data and materials for this manuscript are already published in public databases.

## Abbreviations

DAC: Dystrophin associated complex
AQP4: Aquaporin 4
CSF: Cerebrospinal fluid
SCN: Suprachiasmatic nucleus
CT: Circadian Time
