# Supplementary material for: Circadian timing of the dystrophin associated complex across the brain and body
Source: Fluids Barriers CNS. 2026 Feb 12;23:44. doi: 10.1186/s12987-026-00772-y (PMC12998119; doi:10.1186/s12987-026-00772-y)
Supplement: Supplementary file 1 — Supplementary Material 1 [file 12987_2026_772_MOESM1_ESM.docx]

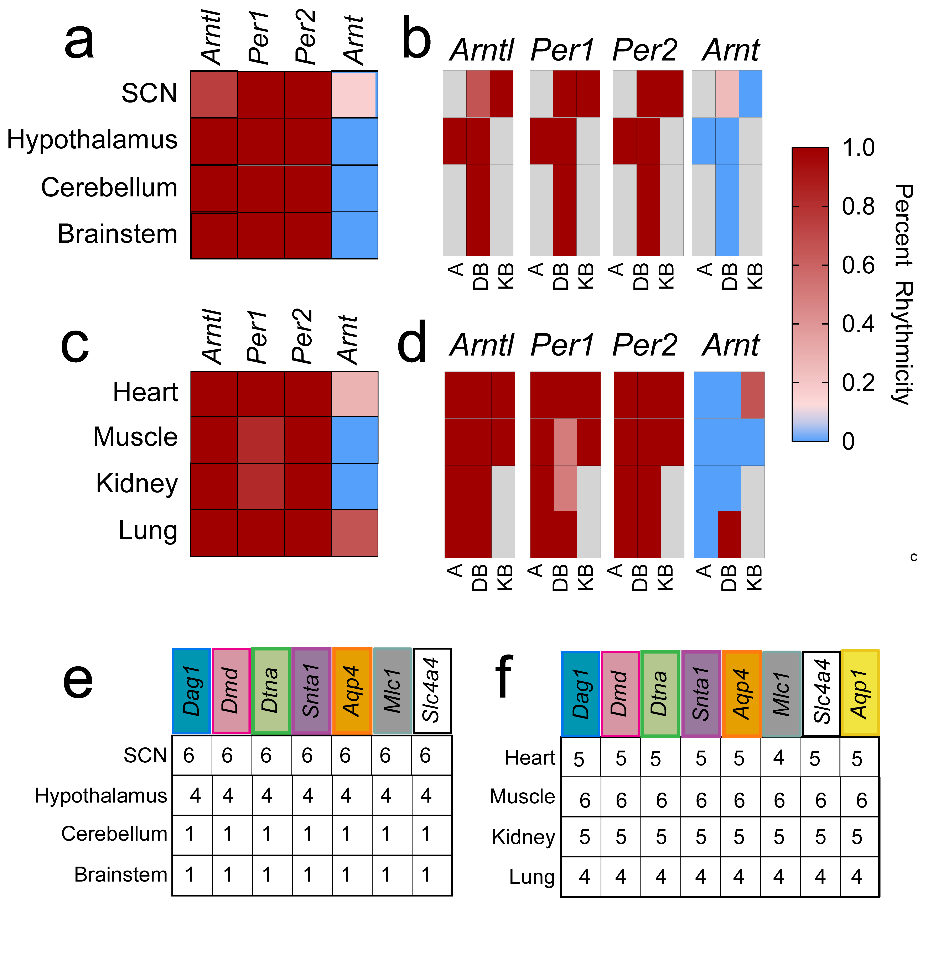
**Supplementary Figure**

**Figure S1. Expression of *Arntl, Per1, Per2* but not *Arnt* exhibits circadian rhythmicity across the brain and body.** (a) Percent rhythmicity of different genes averaged across databases in specific brain regions. (b) The data from (a) broken down by database. A, CircaAge; DB, CircaDB; KB, CircaKB. Gray boxes indicate no results from the database. (right) Percent rhythmicity scale bar. (c) Same as (a), but in different tissues throughout the body. (d) Same as (b) but for body tissues. (e,f) Number of time courses included per tissue type per gene.
